# Tracing the oomycete pathogen *Saprolegnia parasitica* in aquaculture and the environment

**DOI:** 10.1038/s41598-022-16553-0

**Published:** 2022-10-05

**Authors:** Dora Pavić, Dorotea Grbin, Sandra Hudina, Uršula Prosenc Zmrzljak, Anđela Miljanović, Rok Košir, Filip Varga, Josip Ćurko, Zoran Marčić, Ana Bielen

**Affiliations:** 1 Department of Biochemical Engineering, Faculty of Food Technology and Biotechnology, University of Zagreb, 10000 Zagreb, Croatia; 2 Department of Biology, Faculty of Science, University of Zagreb, 10000 Zagreb, Croatia; 3 Labena Ltd, BIA Separations CRO – Molecular Biology Laboratory, 1000 Ljubljana, Slovenia; 4 Department of Seed Science and Technology, Faculty of Agriculture, University of Zagreb, 10000 Zagreb, Croatia; 5 Centre of Excellence for Biodiversity and Molecular Plant Breeding, CoE CroP-BioDiv), 10000 Zagreb, Croatia; 6 Department of Food Engineering, Faculty of Food Technology and Biotechnology, University of Zagreb, 10000 Zagreb, Croatia

**Keywords:** Microbiology techniques, Microbial ecology, Pathogens, Water microbiology, Fungal ecology, Microbial ecology

## Abstract

*Saprolegnia parasitica* causes saprolegniosis, a disease responsible for significant economic losses in aquaculture and declines of fish populations in the wild, but the knowledge of its distribution and prevalence in the environment is limited. We developed a fast, sensitive and specific *S. parasitica* droplet digital PCR (ddPCR) assay and demonstrated its applicability for the detection and quantification of the pathogen in environmental samples: swab DNA collected from the host (trout skin, surface of eggs) and environmental DNA extracted from water. The developed assay was used to assess how abiotic (i.e. physico-chemical parameters of the water) and biotic (health status of the host) factors influence the *S. parasitica* load in the environment. The pathogen load in water samples was positively correlated with some site-specific abiotic parameters such as electrical conductivity (EC) and calcium, while fluorides were negatively correlated, suggesting that physico-chemical parameters are important for determining *S. parasitica* load in natural waters. Furthermore, skin swabs of injured trout had significantly higher pathogen load than swabs collected from healthy fish, confirming that *S. parasitica* is a widespread opportunistic pathogen. Our results provide new insights into various environmental factors that influence the distribution and abundance of *S. parasitica*.

## Introduction

Oomycete pathogens cause diseases in a wide range of plant and animal species, both in the wild and cultured environments, and threaten biodiversity and food security worldwide^1,2^. One of the most destructive oomycete pathogens in freshwater ecosystems is *Saprolegnia parasitica* (Coker, 1923). It causes saprolegniosis, a disease that mainly affects salmonids, from eggs to adult fish, but also other fish species, as well as amphibians, crayfish and other hosts^3,4^. Existing studies on *S. parasitica* mostly focus on its negative impacts in aquaculture: it is responsible for significant economic losses in salmonid farms and hatcheries worldwide^3,5^, and aquaculture facilities can act as pathogen pools from which *S. parasitica* spreads to natural environments^6,7^. In comparison, the potential negative impacts of *S. parasitica* on wild populations of salmonids and other animals remain largely unexplored, although declines in wild salmon populations caused by saprolegniosis have been reported^8,9^.

Existing knowledge on the abiotic and biotic factors influencing the incidence and spread of *S. parasitica* comes either from aquaculture research focusing on host health^10^ or from in vitro microbiological studies^11,12^. Stressful conditions commonly encountered in aquaculture facilities (e.g. temperature shock, infection by bacteria and fungi, stress due to overcrowding or injury) can weaken the host's immune system and increase its susceptibility to *S. parasitica*^10,13^. On the other hand, laboratory studies have shown that zoospore production decreases when water temperature rises above 20 °C^14,15^, while their germination is inhibited under acidic conditions (pH < 4^15^). Humic substances, which constitute the majority of dissolved organic matter in oligotrophic freshwater ecosystems, can inhibit mycelial growth of the pathogen^16^, while Ca^2+^ ions regulate processes such as adhesion, encystment and germination^11,17^. Despite numerous laboratory studies, knowledge about the prevalence of *S. parasitica* in the natural environment is very limited^9,18–20^, making it impossible to define the pathogen's distribution range and analyse ecological parameters that could influence its occurrence. Determining the relationship between the ecological and physico-chemical status of natural waters and the occurrence of *S. parasitica* is central to any attempt to predict the possibility of disease outbreaks in a realistic natural setting.

Currently, the biggest obstacle to such studies is the lack of a fast, effective, sensitive and non-invasive monitoring tool for *S. parasitica*. Traditional *S. parasitica* diagnostic procedures are usually performed after the disease outbreak and are laborious and invasive, requiring the capture and culling of host animals^12,21,22^. Pathogen identification further relies on growing isolates in pure culture and then sequencing their ITS region^6,23,24^. In recent decades, environmental DNA (eDNA) has been increasingly used to detect pathogens in aquatic environments to circumvent invasive and labour-intensive standard methods^25–28^. The target species can be detected in eDNA samples by specific primers designed to amplify only the marker DNA region of the taxon of interest by standard polymerase chain reaction (PCR) or loop-mediated isothermal amplification (LAMP). For example, a LAMP assay has recently been developed that enables highly sensitive and rapid on-site detection of the genus *Saprolegnia*^29^. However, sensitive techniques that allow quantification are often more appropriate, such as quantitative PCR (qPCR) or droplet digital PCR (ddPCR), as they allow monitoring of the dynamics of target species in the environment^26,30–33^. Droplet digital PCR is a relatively new technology and is considered advantageous over the more traditional qPCR since it allows absolute quantification of target DNA without the need for standards and is less susceptible to inhibitors^34–36^. qPCR assay for detection of *Saprolegnia parasitica* was recently reported, but it has very low specificity^37^, leaving the need for development of more specific and effective detection methods. Further, ddPCR has been used for the detection and quantification of several oomycete plant pathogens such as *Aphanomyces euteiches*^38^, *Phytophthora infestans*^39^ and *P. nicotianae*^30^, while no ddPCR protocols have yet been established for oomycete pathogens of animals, including *S. parasitica*.

To improve the knowledge on environmental factors that might influence the distribution and abundance of *S. parasitica* in the environment, in the scope of this study we have: (i) developed *S. parasitica*-specific ddPCR assay; (ii) analysed the effect of different physico-chemical parameters of water on the presence and load of *S. parasitica* in the natural environment; and (iii) analysed the effect of host health status on the *S. parasitica* load.

## Results

### ddPCR assay for the detection of *Saprolegnia parasitica*

**Figure 1:**
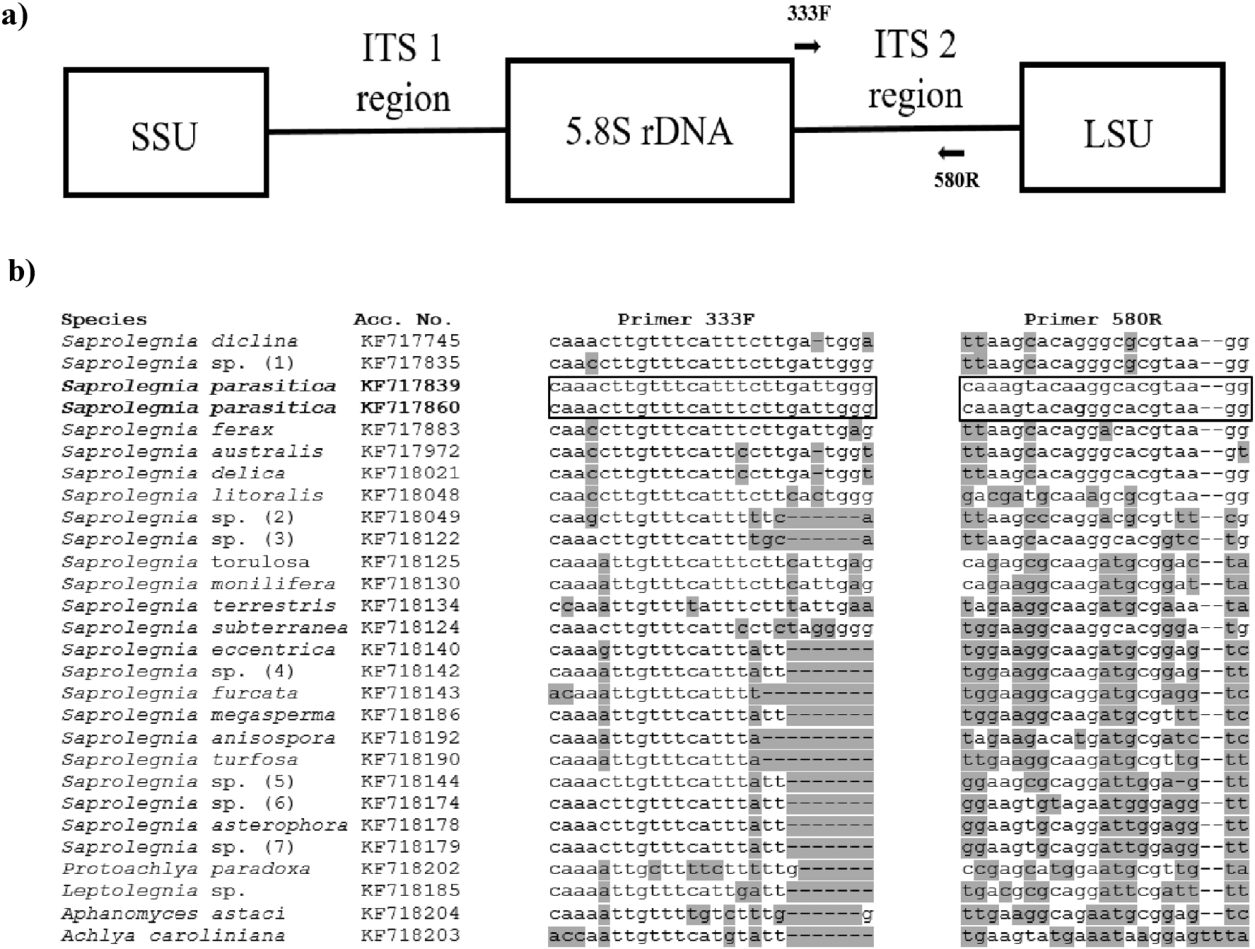
(**a**) Position of *Saprolegnia parasitica*-specific primers 333F and 580R in the internal transcribed spacer (ITS) region (including ITS 1, 5.8S rDNA, and ITS 2) between small subunit (SSU) and large subunit (LSU) rDNA. (**b**) Multiple sequence alignment (MSA) of primer sequences and ITS 2 segments of a range of *Saprolegnia* spp. and other oomycetes. Primer sequences are framed, with a degenerate position (A/G) in the primer 580F marked in bold. Nucleotides that differ between *S. parasitica* and other species within a particular MSA column are shaded in grey. Acc. No.—Genbank accession numbers.

**Figure 2:**
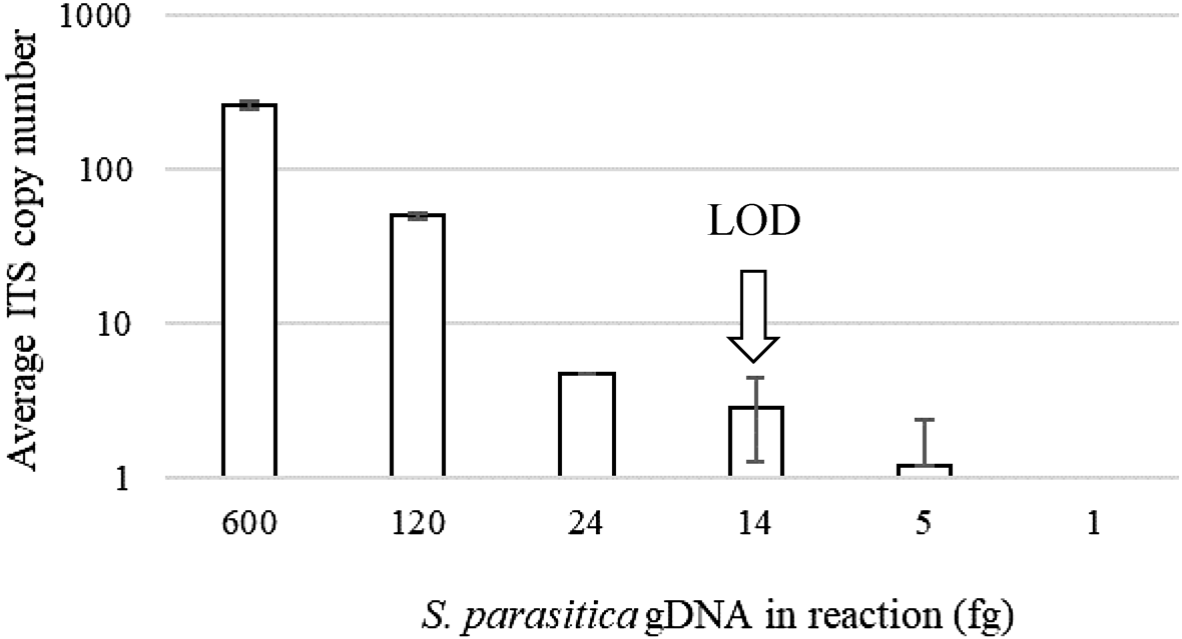
Relation of ITS copy number determined by ddPCR and quantity of *S. parasitica* gDNA (in fg per reaction). Limit of detection (LOD) is marked by an arrow. Error bars represent standard deviation (n ≥ 3).

The assay was developed to target the internal transcribed spacer region 2 (ITS 2), a common marker sequence for *S. parasitica* and other oomycetes^40–42^ (Fig. 1a). The primer pair 333F and 580R was designed to be *S. parasitica*-specific (Fig. 1b), which was confirmed in silico using the Primer-BLAST tool^43^ and in vitro using gDNA from *S. parasitica* and closely related non-target species (Table 1). Four different *S. parasitica* isolates yielded > 10,000 ITS copies per ng gDNA, while gDNA from non-*S. parasitica* oomycetes, including the closely related species *S. diclina* and *S. ferax*, and trout/crayfish gDNA did not yield positive droplets. *Saprolegnia* sp. 1 gDNA was the only exception and yielded ~ 6 ITS copies per ng gDNA. In addition, the sensitivity of the assay was tested using a dilution series of *S. parasitica* gDNA (Fig. 2). The limit of detection (LOD), i.e. the lowest concentration of pathogen DNA that could be reliably detected with the developed assay conditions, was estimated to be 14 fg *S. parasitica* gDNA per reaction (with 91.3% confidence level).

**Table 1:** Specificity of the assay. The *S. parasitica* isolates yielded > 10,000 ITS copies per ng of genomic DNA (marked with +), while the other tested species yielded no ITS copies (no positive droplets, marked with −), except for *Saprolegnia* sp. 1 (SAP-3) that yielded 6 ITS copies per ng of genomic DNA.

Species	Isolate/code	Amplification with primers 333F/580R	Source/reference of the isolate/tissue
*Saprolegnia parasitica*	BF1	+	Pavić et al. ^6^
*Saprolegnia parasitica*	BF2	+	Pavić et al.^6^
*Saprolegnia parasitica*	Z42	+	Pavić et al.^6^
*Saprolegnia parasitica*	Z46	+	Pavić et al.^6^
*Saprolegnia australis*	Z25	−	Pavić et al.^6^
*Saprolegnia delica*	BF5	−	Pavić et al.^6^
*Saprolegnia diclina*	SAP-1	−	Provided by J. Diéguez-Uribeondo
*Saprolegnia ferax*	Z106	−	Pavić et al.^6^
*Saprolegnia litoralis*	SAP-2	−	Provided by J. Diéguez-Uribeondo
*Saprolegnia* sp. 1	SAP-3	−	Provided by J. Diéguez-Uribeondo
*Aphanomyces astaci*	PEC8	−	Provided by F. Grandjean
*Pythium* sp*.*	VU3 3	−	Pavić et al.^6^
*Oncorhynchus mykiss*	T-DNA	−	Provided by E. Teskeredžić
*Pacifastacus leniusculus*	C-DNA	−	Provided by I. Maguire

Next, the applicability of the developed ddPCR assay for quantifying *S. parasitica* load in environmental DNA (eDNA) samples was demonstrated using trout eggs infected with *S. parasitica* in the laboratory. Swab samples taken from the infected eggs had a significantly higher *S. parasitica* load (average 6,195 ITS copies per ng of total eDNA, min = 63, max 18,148) than healthy eggs from the hatchery (average 1.5 ITS copies per ng of total eDNA, min = 0, max 7; Mann–Whitney U test, W = 0, p < 0.01) and healthy eggs used as negative controls in the infection trial (average 2.2 ITS copies per ng of total eDNA, min = 0, max 9; Mann–Whitney U test, W = 0, p = 0.01). No significant difference in *S. parasitica* load was found between the healthy eggs from the hatchery and the healthy eggs used as negative controls in the infection trial (Mann–Whitney U test, W = 12, p = 1).

### Variations of *Saprolegnia parasitica* load in the environment

We used the newly developed ddPCR assay to analyse the effect of water composition and health status of the host on *S. parasitica* load in water and on the skin surface of its fish hosts, respectively.

### Effect of physico-chemical parameters of water quality on *Saprolegnia parasitica* load in the water

**Figure 3:**
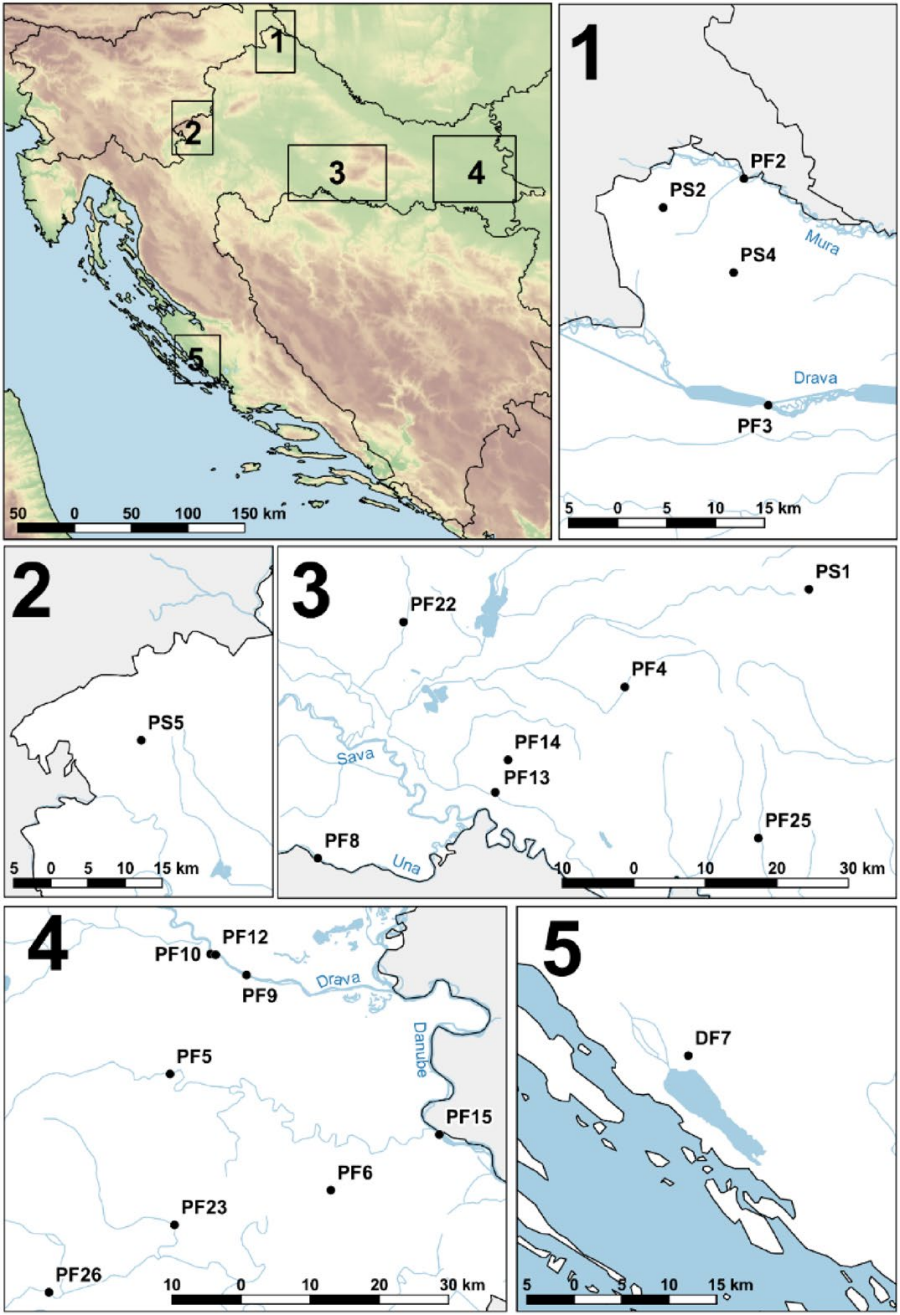
Positions of 21 water sampling locations in Croatia. P—Pannonian region, D—Dinaric region, F—lotic water system (flowing), S—lentic water system (stagnant). The map was generated using QGIS 3.10.7.

We collected water samples from stagnant and flowing water bodies in Croatia (Fig. 3, Supplementary Table S1) and *S. parasitica* was detected in 13 of 21 water samples (62%). The average *S. parasitica* load in water samples was 3.24 ITS copies per ng of total eDNA (min = 0, max 14). Physico-chemical analyses showed overall good quality of collected water samples, but some samples exceeded legal limits (Official Gazette, 96/2019): nitrates were elevated in 6 out of 21 samples (28.6%), ammonium in 2/21 (9.5%) and total phosphorus in 2/21 (9.5%) (Supplementary Table S2).

**Figure 4:**
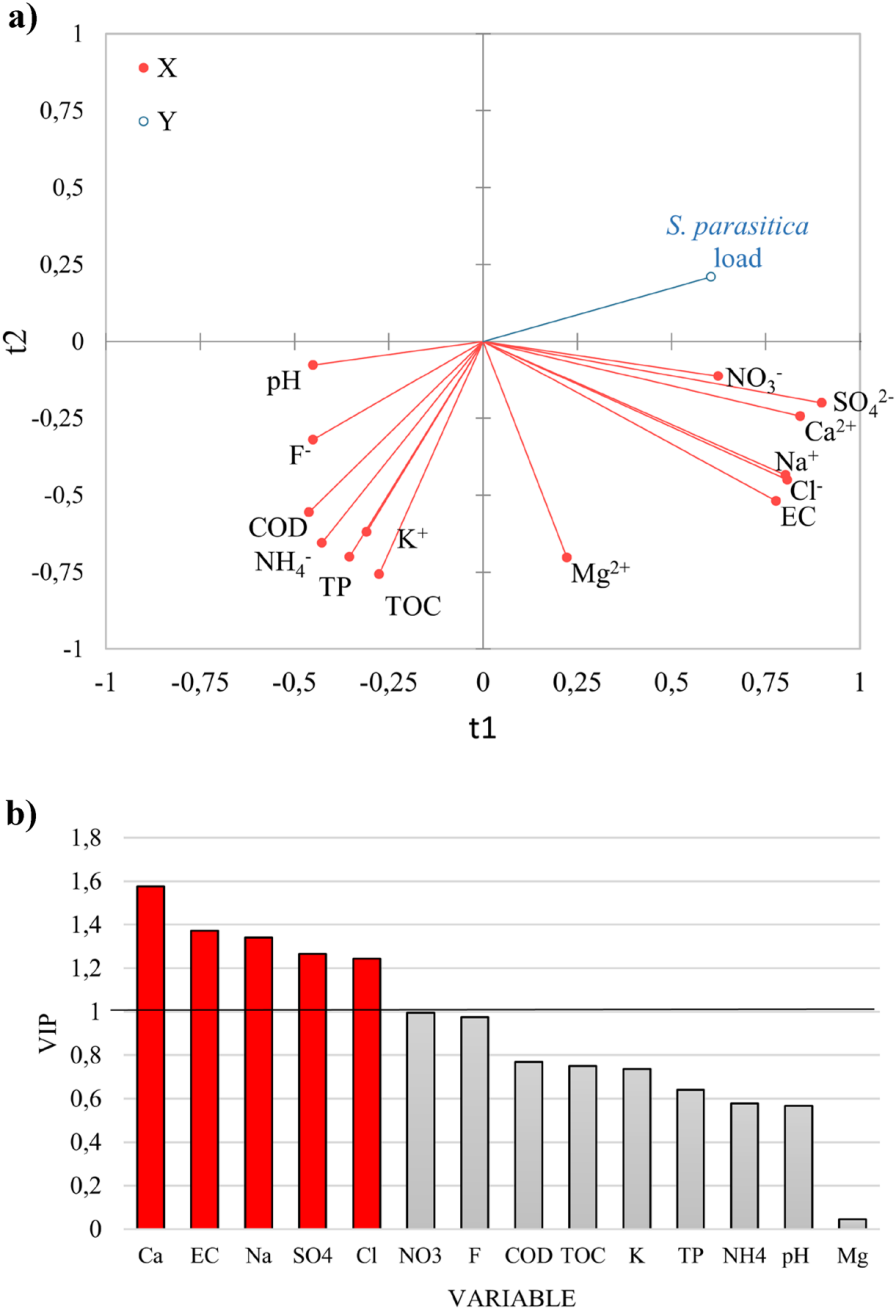
(**a**) Correlation radar describing the relationship between *S. parasitica* load (response variable, Y, blue line) and the physico-chemical parameters of the water (explanatory variables, X, red lines). The percentages of variances in X and Y explained by each variable are indicated on the respective axes. (**b**) The variable importance in projection (VIPs) for explanatory variables of the first component (t1). VIPs > 1 indicate the explanatory variables that contribute most to the PLS model, while VIPs < 0.8 contribute little.

Using the PLS-R modelling, we analysed the relationship between *S. parasitica* load (response variable, Y) and various physico-chemical parameters of the water (explanatory variables, X). As for the model quality indices R^2^X and R^2^Y in component 1, 35% of the variance in the set of explanatory variables (X) was used to explain 37% of the variance for the response variable (Y). In component 2, 61% of the set of explanatory variables (X) was used to explain 41% of the response variable (Y). Q^2^, as a measure of goodness of prediction, showed that component 1 contributed 15% and component 2 contributed 5% to model quality. The relationship between blocks of predictor and response variables is visually represented in the form of a correlation radar (Fig. 4a), with positively correlated variables close together and negatively correlated ones far apart. Multivariate PLS-R analysis showed that Ca^2+^ (r = 0.56) and EC (r = 0.49) were the most important parameters that positively influenced the *S. parasitica* load in the water samples, followed by Na^+^ (r = 0.47), SO_4_^2−^ (r = 0.45), Cl^−^ (r = 0.44) and NO_3_^−^ (r = 0.35), while the most pronounced negative correlation was found for F^−^ (r = − 0.35), followed by COD (r = − 0.27), TOC (r = − 0.27), K^+^ (r = − 0.26), TP (r = − 0.23), NH_4_^+^ (r = − 0.21) and pH (r = − 0.20) (Fig. 4a, Supplementary Table S3). Ca^2+^, EC, Na^+^, SO_4_^2−^ and Cl^−^ also had VIP values > 1, meaning that they are considered highly relevant in explaining the *S. parasitica* load in water and contribute significantly to the model^44,45^ (Fig. 4b).

### Effect of host health status on *Saprolegnia parasitica* load on the trout skin

We also inspected whether the health status of the trout host influences *S. parasitica* skin load. At the trout farms, we sampled animals (brown trout, *Salmo trutta* Linnaeus, 1758, and rainbow trout, *Oncorhynchus mykiss* Walbaum, 1792) that were apparently healthy as well as animals that had skin injuries, but without gross signs of saprolegniosis (Supplementary Table S4). No significant differences in *S. parasitica* load were found between rainbow and brown trout (data not shown), and thus data from both species were pooled together in subsequent analyses. The results have shown that the health status of the host significantly influenced *S. parasitica* skin load, and swab samples of injured trout had significantly higher *S. parasitica* load (on average 9154 ITS copies per ng of total eDNA; min = 0, max 118,094) than the apparently healthy specimens (average 1.1 ITS copies per ng of total eDNA; min = 0, max 11; Mann–Whitney U test, W = 27, p < 0.001, Supplementary Table S4). Noteworthy is the fact that one fish with skin lesions (B10) was *S. parasitica* negative.

## Discussion

The developed *S. parasitica*-specific ddPCR assay is highly sensitive and allows absolute quantification of pathogen DNA in the environment, enabling for the first time rapid and simple monitoring of the pathogen. In this study, we have demonstrated its applicability in *S. parasitica* monitoring in the environment and in aquaculture. Most importantly, our results provide insights into the environmental factors that influence the abundance of the pathogen in different types of freshwater ecosystems.

We designed *S. parasitica*-specific primers targeting the ITS region of rDNA, a standard high copy number nuclear marker for oomycetes that is abundantly represented in DNA databases^42,46,47^. We maximised the specificity of the assay by selecting *S. parasitica*-specific primer sequences from the regions of highest divergence with closely related *Saprolegnia* species and other oomycetes, and by using touchdown PCR. This is a step forward in comparison to the previously reported qPCR assay for the detection and quantification of *S. parasitica*^37^ that also targeted the ITS region, but with very low specificity: the specificity was tested with only two oomycete species, *S. diclina* and *A. astaci*, and the selected primers were not discriminatory for closely related species, i.e. the forward primer had the identical sequence to *S. australis, S. delica, S. diclina, S. ferax* and *Saprolegnia* sp*.* 1 among many others, while the reverse primer differed from related species by only one or two bp. Our assay showed high discrimination between *S. parasitica* (> 10,000 copies per ng gDNA) and closely related species (no amplification). Only one closely related non-target species, *Saprolegnia* sp. 1 (one bp difference along the 333F primer), was amplified but yielded only one positive droplet per reaction (or ~ 6 ITS copies per ng gDNA), which was considered a false positive. *Saprolegnia* sp. 1 was rarely detected during sampling at salmonid fish farms and hatcheries^23,24^, indicating a low risk of false positives in ddPCR monitoring of *S. parasitica*. Furthermore, the sensitivity of the ddPCR method developed here, with LOD of 14 fg *S. parasitica* gDNA per ddPCR reaction (~ 3 ITS copies), is high and comparable to or more sensitive than other published qPCR/ddPCR assays for oomycetes^30,39,48^. Although the ITS copy number per *S. parasitica* genome is not known, considering the *S. parasitica* genome size of 63 Mb^49^, 14 fg corresponds to about 0.2 genomic units and implies that the copy number of *S. parasitica* ITS should be about 10, at least for isolate BF1 used here. This is in line with available data for other oomycete species: the ITS copy number varies between several tens and several hundreds between different oomycete species, but also between different isolates of the same species^30,38^.

We used the developed assay as a non-invasive monitoring approach and showed that *S. parasitica* is ubiquitous in the environment, as we detected it in a number of water samples (62% of analysed samples), on the surface of trout eggs and adult trout from aquaculture facilities, and on the surface of the crayfish exoskeleton in the wild (unpublished results for signal crayfish *Pacifastacus leniusculus* Dana, 1852 and narrow-clawed crayfish *Pontastacus leptodactylus* (Eschscholtz, 1823), previously reported as carriers/hosts of *S. parasitica*^50,51^). The developed method allowed us to gain insights into the ecology of the pathogen in relation to biotic and abiotic parameters in both wild populations and aquaculture. In aquaculture, for example, trout with injuries (i.e. skin lesions) had significantly higher *S. parasitica* skin loads than the healthy specimens. *Saprolegnia parasitica* was detected in 91% of swabs from injured fish, indicating that it is a dominant opportunistic skin pathogen of trout. Only one swab sample from trout with injuries (B10) was *S. parasitica*-negative, presumably because another pathogen has outcompeted *S. parasitica*. In contrast, *S. parasitica* was mostly not detected (or its load was low) in the skin swabs of apparently healthy adult trout and trout eggs. Overall, our results show that *S. parasitica* is ubiquitous in fresh waters (both in the water column and on the host surface) and can infect stressed, immunocompromised or injured host individuals at any time, as previously reported^52,53^.

By combining absolute quantification of the pathogen with the data on physico-chemical properties of natural waters, we found for the first time a correlation between *S. parasitica* load and some water parameters. The positive correlation was strongest for Ca^2+^ and EC, while the parameter with the strongest negative influence was F^−^. Calcium concentrations in water samples within our dataset ranged from 2.15 to 119.4 mg/L, with a median of 37.1 mg/L, which is higher than the global freshwater median of 4 mg/L^54^. Based on in vitro microbiological studies, calcium ions may positively influence the developmental stages and infection process of oomycetes^11,17,55^. For example, in *S. parasitica*, the number and length of long hooked hairs on cysts increased after the addition of 5550 mg/L CaCl_2_, enhancing the adhesion of cysts to the host surface^11^. Our results show for the first time that higher than average Ca^2+^ concentrations in surface waters, which are common in karst areas^56^, favour the growth/development of *S. parasitica*. This is in line with the above-mentioned in vitro studies showing the positive effect of (even) higher concentrations of calcium ions expected when the pathogen invades host tissues^57,58^.

In addition, electrical conductivity, which is related to the total ion content of the water and mainly to the sodium, chloride and calcium ions’ concentration, was found to correlate positively with *S. parasitica* load. Sodium chloride in high concentrations (> 1000 mg/L) is used as an effective and non-toxic method to control *Saprolegnia* sp. as it can reduce the vegetative growth of the pathogen and the formation, release and proliferation of sporangia^59^. In freshwater ecosystem sodium and chloride concentrations vary from country to country. For example, the range of groundwater limits set by European Union countries for chloride is between 24 and 12,300 mg/L, while limits for sodium in drinking water are between 50 and 450 mg/L^60^. In our study, water samples with *S. parasitica* load > 5 ITS copies/ng had higher average chloride (40.2 mg/L) and sodium (1.3 mg/L) concentration than the water samples with lower *S. parasitica* load (17.9 and 0.6 mg/L, respectively). Our results thus suggest that moderate salt concentrations in natural waters may have a positive effect on *S. parasitica*, which is also consistent with the fact that sodium and chloride concentrations are higher in host tissues than in water^61^.

Fluoride concentration was the most important factor that negatively correlated with *S. parasitica* load in water, although it did not contribute significantly to the model (VIP < 1). It is known that excessive fluoride concentrations in the environment can affect microbial communities due to its negative effects on microbial physiology^62,63^. The World Health Organization (WHO) limit for fluoride concentrations in drinking water is 1.5 mg/L, while fluoride concentrations in unpolluted freshwaters range from 0.01 to 0.3 mg/L^64^. In our dataset, water samples with *S. parasitica* load > 5 ITS copies/ng had an average of 0.38 mg F^−^/L, compared to 0.47 mg/L in the water samples with lower *S. parasitica* loads. To our knowledge, there are no data on the toxicity of fluoride to oomycetes, but this result suggests an inhibitory effect of environmentally relevant fluoride concentrations on *S. parasitica*. However, further studies, including more extensive monitoring, are needed to confirm this since the negative correlation of fluorides and *S. parasitica* load in water was not significant in our study.

Overall, we have shown that the newly developed ddPCR method allows sensitive detection and quantification of *S. parasitica* load in various environmental DNA samples and avoids the qualitative and labour-intensive cultivation used so far. The novel method could be used in salmonid aquaculture to monitor variations in *S. parasitica* loads in both skin/egg swabs and water. Currently, toxic antioomycetic chemicals are continuously used to prevent disease outbreaks, regardless of the actual load of the pathogen. If outbreaks could be predicted in a timely manner through ddPCR-based monitoring, the use of antioomycetic chemicals could be adjusted to the current pathogen load. In this way, both chemical pollution and pathogen transmission to downstream waters could be reduced. Here, we have used the developed method to gain new insights into the ecological requirements of the pathogen and provide a basis for identifying natural habitats at increased risk for outbreaks of *S. parasitica*. For example, environmentally relevant Ca^2+^ and EC levels were found to have a positive effect on *S. parasitica* load, while F^−^ had a negative effect. However, more extensive field monitoring is needed to confirm and support our conclusions. In addition, laboratory experiments are needed to evaluate how the selected water parameters affect the virulence of the pathogen by examining various experimental endpoints such as sporulation efficiency, zoospore germination, host mortality, etc.

## Methods

### Samples used for assay development and validation

For the development of the assay (i.e. to analyse the sensitivity and specificity of the designed primers), we used mycelia from pure cultures of *S. parasitica* (positive control) and other oomycetes as well as tissue samples from known host/carrier species, namely the skin of healthy rainbow trout (*Oncorhynchus mykiss*) and the abdominal cuticle of the signal crayfish (*Pacifastacus leniusculus*)^50,53^ (Table 1). Oomycete mycelia were grown in liquid glucose-yeast extract (GY) medium^65^ for two days at 18 °C, then washed with sterile distilled water and centrifuged at 10,000×*g* for 15 min^6^. The resulting pellets (approx. 30 mg wet weight per sample) were stored at − 20 °C until DNA extraction^6^.

For assay validation, we used environmental water samples collected at 21 different locations in Croatia (Fig. 3, Supplementary Table S1), as well as samples from aquaculture (trout farms), i.e. 45 swabs collected from trout skin and eggs (rainbow trout, *O. mykiss*, and brown trout, *S. trutta*) (Supplementary Table S4).

Water samples were collected in winter 2018/2019 (Supplementary Table S1, Fig. 3) into autoclaved polyethylene bottles washed three times with the water sample before filling and then kept in the dark and on ice during transport. Then, 500 mL of water per sampling location for determination of physico-chemical parameters of the water was freezed at − 20 °C, until the analyses of pH, electrical conductivity (EC), NH_4_^+^, NO_3_^−^, SO_4_^2−^, F^−^, Cl^−^, Na^+^, K^+^, Mg^2+^, Ca^2+^, total organic carbon (TOC), total phosphorus (TP) and chemical oxygen demand (COD) (Supplementary Table S2 and Supplementary Methods). For DNA extraction, 2 L of the water per sampling location was filtered immediately upon return to the laboratory through a 3-branch stainless steel manifolds (Sartorius, Germany) using hydrophilic sterile polyethersulfone filters (d = 47 mm, pore size = 0.22 µm; Millipore Express® PLUS, Germany). Filters with the microbial biomass were stored at − 20 °C until DNA extraction.

Swab samples containing the epibiotic community of *S. parasitica* hosts (including *S. parasitica*, if present) were collected from adult trout (N = 30) and trout eggs (N = 15) sampled in four selected trout farms in Croatia: Gračani (with rainbow trout *O. mykiss*), Kostanjevac (with brown trout *S. trutta*), Radovan (*O. mykiss*) and Solin (*O. mykiss*), in winter 2018/2019 (Supplementary Table S1). After capture at the farm, each animal was transported separately in a plastic bag or container and taken directly to the laboratory, where swab samples were collected as described in Pavić et al.^26^.

Live, healthy, fertilised trout eggs were collected at trout farms. About 30–50 eggs were placed in a sterile 50 mL Falcon tube filled with farm water and this constituted one sample. Since no *S. parasitica*-infected eggs were available at the trout farms at the time of sampling, some of the collected egg samples were infected with *S. parasitica* in the laboratory. A total of 15 egg samples were collected: five samples of healthy eggs analysed directly after collection at the farms, five samples of healthy eggs used as negative controls in a laboratory infection trial, and five egg samples infected with *S. parasitica* in the laboratory (Supplementary Table S4). Infection of the eggs with *S. parasitica* and collection of the epibiotic community from the surface of the eggs (infected and healthy) was performed according to Liu et al.^66^ with some modifications (details in Supplementary Methods).

No permissions were needed for the experimental work with trout eggs and adult trout performed within this study. Regarding the experimental infection of fertilized trout eggs with *S. parasitica*, no permission was needed since the infection experiment lasted up to 1 week post-fertilization. According to the EU Directive 2010/63/EU^67^ on the protection of animals used for scientific purposes, the early life stages of vertebrates, including fish, are not protected as animals until being capable of independent feeding. For trout, the first independent feeding typically takes place about 8 weeks’ post-fertilization, and our experiment was terminated much earlier. Thus, the infection experiment does not fall into the regulatory frameworks dealing with animal experimentation (including ARRIVE guidelines). Regarding the adult trout, permissions were not needed to perform the sampling as animals were already dead at the beginning of sampling and were collected as a part of routine harvesting at the fish farms.

### DNA extraction

DNA from (i) pure culture oomycete mycelia (listed in Table 1), (ii) pellets of epibiotic communities from the surface of trout skin and eggs, and (iii) filters containing microbial communities from water was extracted using the NucleoSpin® Microbial DNA Kit (Macherey Nagel, Germany) according to the protocol provided by the manufacturer with minor modifications. Samples were lysed by shaking (medium strength, 20 min) on a Vortex Mixer (Corning, USA) using Macherey Nagel Bead Tubes type B. DNA was eluted from the column using the initial 100 µL eluate for a second elution to increase DNA yield and concentration of the final sample. The NucleoSpin® Tissue Kit (Macherey Nagel, Germany) was used to extract genomic DNA from trout and crayfish, following the protocol provided. The quantity and quality of DNA samples was assessed by agarose gel electrophoresis and QuantiFluor ONE dsDNA Dye on a Quantus Fluorometer (Promega, Germany).

### Design of *S. parasitica*-specific primers

ITS sequences of *S. parasitica* and a number of *Saprolegnia* spp. and other oomycetes (Supplementary Table S5) were selected based on an available study on the molecular taxonomy of *Saprolegnia*^42^. Sequences were retrieved from the National Center for Biotechnology Information (NCBI) database using the Batch Entrez tool (https://www.ncbi.nlm.nih.gov/sites/batchentrez) and aligned using MAFFT^68.^ Alignment was edited in SeaView^69^ and BioEdit^70^. Potential *S. parasitica*-specific primer sequences were selected after manual inspection of the alignment and positioned in the region of maximum divergence to other closely related *Saprolegnia* spp. (Fig. 1b): forward primer 333F (5' CAA ACT TGT TTC ATT TCT TGA TTG GG 3') and reverse primer 580R (5' CCT TAC GTG CCY TGT ACT TTG 3') amplifying a *S. parasitica* DNA segment of 247 bp.

### Droplet digital polymerase chain reaction (ddPCR)

The ddPCR assay was performed using the QX200™ Droplet Digital™ PCR System (Bio-Rad, USA). Droplet digital PCR reactions and preparations were performed in a dedicated pre-PCR room and PCR hood, separated from both the DNA extraction room and the post-PCR room. Each reaction mixture contained 10 µL of 2 × QX200™ ddPCR™ EvaGreen® Supermix, 200 nM forward and reverse primers, DNA template (1 µL gDNA, 4 µL swab DNA and 8 µL filter DNA) and DNase/RNase-free H_2_O in a total volume of 20 µL. These reaction mixtures were mixed with 70 µL of droplet generation oil and droplets were generated using the QX200™ droplet generator, and then transferred to 96-well PCR plates to perform PCR amplifications using the C1000 Touch Thermal Cycler.

Amplification conditions were developed starting with the melting temperatures (Tm, salt-adjusted) of the primers predicted using the OligoCalc tool (http://biotools.nubic.northwestern.edu/OligoCalc.html): 62 °C for 333F and 60 to 61 °C for 580R. Thus, during the assay development, we have tested annealing temperatures of 58 °C, 60 °C and 63 °C. Annealing at 60 °C gave optimal results with *S. parasitica* gDNA template, but at this temperature several related species also yielded some positive droplets (data not shown). Therefore, we adopted a touchdown cycling protocol starting with annealing at 63 °C and ending at 60 °C as follows: denaturation at 95 °C for 5 min, followed by 15 cycles of 95 °C for 30 s and 63 °C for 1 min, followed by another 30 cycles of 95 °C for 30 s and 60 °C for 1 min. Finally, the signal stabilisation step was performed at 4 °C for 5 min and 90 °C for another 5 min, with a final hold at 4 °C.

After the PCR reaction, the droplets were checked for fluorescence using the QX200 Droplet Reader and the data were analysed using QuantaSoft™ version 1.7.4. Four positive controls (genomic DNA from *S. parasitica* pure culture) and one negative control (no template control, NTC) were included in the specificity and sensitivity assays, while one positive control and one NTC were used in the analyses of filters and swabs. Only samples with more than 10,000 droplets were used for the analysis. The limit of detection (LOD) of the ddPCR assay was determined using a serial fivefold dilution of *S. parasitica* gDNA, starting with a concentration of 0.06 ng/µL. Each dilution was tested in triplicate.

Next, we applied the developed *S. parasitica* ddPCR detection assay to analyse the pathogen load in environmental DNA (eDNA) extracted from 21 water samples and 45 trout skin/egg biofilm samples (Supplementary Tables S1 and S2). To obtain a comparable quantification of *S. parasitica* between eDNA samples of different origin/type, the pathogen load was expressed as the number of *S. parasitica* ITS copies per ng of total eDNA.

### Data analyses

We analysed the effects of physico-chemical properties of water samples (as abiotic environmental parameters) and health status of eggs and adult fish (as biotic parameters) on *S. parasitica* load, as listed in Table 2.

**Table 2:** Analyses of the effect of different variables on *S. parasitica* load.

eDNA sample type for which the *S. parasitica* load was determined	Variable		Number of analysed samples per variable	Statistical analysis
Microbial community from water	Physico-chemical properties of water samples	Values for the following parameters: pH, EC, NH_4_^+^, NO_3_^−^, SO_4_^2−^, F^−^, Cl^−^, Na^+^, K^+^, Mg^2+^, Ca^2+^, TOC, TP and COD	21 sites	Partial least squares regression (PLS-R)
Biofilm of trout eggs	Health status	*S. parasitica* infected or healthy	15 in total 5 laboratory infected 5 healthy infection trial neg. controls 5 healthy from the hatchery	Mann–Whitney U test
Biofilm of adult trout skin collected at the fish farms	Health status	Injured or healthy	30 in total 17 injured 13 healthy	Mann–Whitney U test

Partial least squares regression (PLS-R) approach was used to examine the effects of the water composition variables (explanatory variables, X) on the *S. parasitica* load expressed as the number of *S. parasitica* ITS copies per ng of total eDNA (response variable, Y). The PLS-R analyses were performed using XLSTAT version 2021.3.1.1189 software for data analysis and visualisation (Addinsoft, Microsoft Excel).

Data on *S. parasitica* load in biofilm samples were tested for normality using the Shapiro–Wilk test. As they did not follow a normal distribution, non-parametric Mann–Whitney U test was used to examine correlations and differences in *S. parasitica* load between different sub-groups of biofilm samples. Tests were performed in R v. 3.2.0 (R Core Team, 2020), with significance level set at p = 0.05.

## Supplementary Information

Supplementary Information 1.

Supplementary Tables.

## Data Availability

All data generated or analysed during this study are included in this published article and its Supplementary Information files.
