# Supplementary material for: Tracing the oomycete pathogen Saprolegnia parasitica in aquaculture and the environment
Source: Sci Rep. 2022 Oct 5;12:16646. doi: 10.1038/s41598-022-16553-0 (PMC9534867; doi:10.1038/s41598-022-16553-0)
Supplement: Supplementary file 1 — Supplementary Information 1. [file 41598_2022_16553_MOESM1_ESM.pdf]

Tracing the oomycete pathogen *Saprolegnia parasitica* in aquaculture and the environment

Dora Pavić[1], Dorotea Grbin[1,2], Sandra Hudina[2], Uršula Prosenc Zmrzljak[3], Anđela Miljanović[1], Rok Košir[3], Filip Varga[4,5], Josip Ćurko[6], Zoran Marčić[2], Ana Bielen[1,*]

[1]Department of Biochemical Engineering, Faculty of Food Technology and Biotechnology, University of Zagreb, 10000 Zagreb, Croatia

[2]Department of Biology, Faculty of Science, University of Zagreb, 10000 Zagreb, Croatia

[3]Labena Ltd, BIA Separations CRO – Molecular Biology Laboratory, 1000 Ljubljana, Slovenia

[4]Department of Seed Science and Technology, Faculty of Agriculture, University of Zagreb, 10000 Zagreb, Croatia

[5]Centre of Excellence for Biodiversity and Molecular Plant Breeding (CoE CroP-BioDiv), 10000 Zagreb, Croatia

[6]Department of Food Engineering, Faculty of Food Technology and Biotechnology, University of Zagreb, 10000 Zagreb, Croatia

*Corresponding author.

E-mail address: abielen@pbf.hr (A. Bielen).

Supplementary Methods

Determination of physico-chemical parameters

Physico-chemical parameters, i.e. pH, electrical conductivity (EC), NH_4^+, NO_3^-, SO_4^{2-}, F^-, Cl^-, Na^+, K^+, Mg^{2+}, Ca^{2+}, total organic carbon (TOC), total phosphorus (TP) and chemical oxygen demand (COD) were determined for 21 water samples (Supplementary table S2). EC was measured at 22 °C using a pH / Cond – meter inoLab 720 instrument (WTW GmbH & Co., Germany). The TP in water samples was determined according to ISO 6878-1:1986. Chemical oxygen demand was determined according to ISO 6060:1989. Total organic carbon was determined using Shimadzu TOC-L CPH analyzer (Shimadzu, Japan) according to HRN EN 1484:2002. Anions (fluorides, chlorides, nitrates, and sulfates) and cations (calcium, magnesium, sodium, potassium and ammonium) were determined according to HRN EN ISO 10304-1:2009/cor.1:2012 and HRN EN ISO 14911:2001 using DIONEX DX-500 ion chromatograph (Thermo Fisher Scientific, USA) with a conductometric detector (CD20) in combination with electrochemical suppressor. An anion column Dionex IonPac AS9-HC and a cation column Dionex IonPac CS12A were used. Nine mmol/L Na_2CO_3 was used to elute the anions and 20 mmol/L methanesulfonic acid was used to elute the cations. Both eluent flow rate through the columns were 1 mL/min. The samples were filtered using a 0.45 μm pore size membrane filter prior to manual injection of the samples.

Experimental infection of trout eggs with *S. parasitica* and collection of swabs

Trout eggs corresponding to one sample were placed in a sterile glass cup filled with 200 mL of fish farm water and equipped with air pumps, and kept at 8 - 10 °C. Next, mycelial tips of *S. parasitica* were aseptically transferred from GY agar into a Falcon tube containing autoclaved

hemp seeds in distilled water and incubated at 18 °C for three days until colonisation with *S. parasitica* hyphae became visible. Four hemp seeds overgrown with *S. parasitica* mycelium were then added to cups containing trout eggs, while no hemp seeds were added to the control cups. After seven days, the infected mycelium-covered eggs (or the healthy eggs) were transferred to sterile Falcon tubes filled with 30 mL of sterile phosphate-buffered saline (PBS).

To collect the epibiotic community from the surface of the eggs (infected and healthy), the Falcon tubes containing the eggs with sterile phosphate-buffered saline (PBS) (app. 30 mL) were shaken in an incubator (Innova® 42, New Brunswick™, Germany) at 180 rpm and 6 °C for 12 hours. The eggs were then removed with a sterile spoon and the entire volume of the suspension was left to settle at 4 °C for approximately 30 minutes. Then about 2 mL of the suspension was removed from the bottom of the Falcon tube using a sterile Pasteur pipette and transferred to a microcentrifuge tube (1.5 mL). The suspension was centrifuged at 10 000 x g at 4 °C for 15 minutes. The supernatant was removed and the cell pellets were frozen at - 20 °C until DNA extraction.
