# Supplementary material for: Tracing the oomycete pathogen Saprolegnia parasitica in aquaculture and the environment
Source: Sci Rep. 2022 Oct 5;12:16646. doi: 10.1038/s41598-022-16553-0 (PMC9534867; doi:10.1038/s41598-022-16553-0)
Supplement: Supplementary file 2 — Supplementary Tables. [file 41598_2022_16553_MOESM2_ESM.pdf]

Supplementary Tables

of the manuscript

Tracing the oomycete pathogen *Saprolegnia parasitica* in aquaculture and the environment

Dora Pavić[1], Dorotea Grbin[1,2], Sandra Hudina[2], Uršula Prosenc Zmrzljak[3], Anđela Miljanović[1], Rok Košir[3], Filip Varga[4,5], Josip Ćurko[6], Zoran Marčić[2], Ana Bielen[1,] *

[1]Department of Biochemical Engineering, Faculty of Food Technology and Biotechnology, University of Zagreb, 10000 Zagreb, Croatia

[2]Department of Biology, Faculty of Science, University of Zagreb, 10000 Zagreb, Croatia

[3]Labena Ltd, BIA Separations CRO – Molecular Biology Laboratory, 1000 Ljubljana, Slovenia

[4]Department of Seed Science and Technology, Faculty of Agriculture, University of Zagreb, 10000 Zagreb, Croatia

[5]Centre of Excellence for Biodiversity and Molecular Plant Breeding (CoE CroP-BioDiv), 10000 Zagreb, Croatia

[6]Department of Food Engineering, Faculty of Food Technology and Biotechnology, University of Zagreb, 10000 Zagreb, Croatia

*Corresponding author.

E-mail address: abielen@pbf.hr (A. Bielen).

Table S1 Water samples collected from different locations in Croatia.

Code	Location	HTRS96 coordinate system		Lotic/lentic water system	Sampling period	Copies per µL of original sample	DNA concentration (ng/µL)	*Saprolegnia parasitica* ITS copies/ng of total DNA
		x	y					
PF2	Mura River (Mursko Središće)	495276	5152812	lotic	December 2018	28.60	3.52	8
PF3	Drava River (near Varaždinsko Lake)	497761	5129630		December 2018	0.19	0.48	0.4
PF4	Pakra River (Lipik)	553535	5032272		December 2018	0.93	2.81	0.3
PF5	Vuka River	656960	5035834		January 2019	0	0.47	0
PF6	Bosut River	680237	5019011		January 2019	0.05	2.26	0
PF8	Una River (Slabinja)	510612	5008303		January 2019	3.13	1.70	2
PF9	Drava River (Osijek)	668033	5050185		January 2019	0.11	0.99	0.1
PF10	Karašica River	662818	5053231		January 2019	0.44	5.30	0
PF12	Karašica River (Petrijevci)	663568	5053106		January 2019	0.11	2.59	0
PF13	Dravinja River (Strug)	535413	5017538		January 2019	0.52	3.45	0.2
PF14	Novljančica River (Novska)	537193	5022072		January 2019	13.75	1.10	13
PF15	Dunav River (Vukovar)	695937	5027062		January 2019	3	0.28	11
PF22	stream (Kutinska Slatina)	522589	5041311		January 2019	1.91	0.41	5
PF23	stream (Soljak)	657616	5013986		January 2019	0	1.16	0
PF25	canal (Nova Gradiška)	572189	5011124		January 2019	1.54	0.21	7
PF26	canal (Sredanci)	639434	5004211		January 2019	0	0.42	0
DF7	Vransko Lake main canal	422963	4868401		January 2019	19.25	1.37	14
PS1	Zvečevo Lake	579266	5045889	lentic	December 2018	0.47	0.72	1
PS2	Lapaž Lake	487012	5149847		December 2018	0	0.66	0
PS4	Mačkovec Pond	494205	5143202		December 2018	0	1.97	0
PS5	Vrabac fish farm (Kostanjevac)	420850	5064594		March 2019	3.85	0.62	6

Table S2 Physico-chemical parametres of the collected water samples. Values higher that the limit issued by the Status regulation on the water quality (NN 96/19) are shown in bold.

Code	EC µs/cm	NH4 mg NH4-N/L	NO3 mg NO3-N/L	SO4 mg/L	F mg/L	Cl mg/L	Na mg/L	K mg/L	Mg mg/L	Ca mg/L	TOC mg/L	TP mg P/L	COD mg O2/L	pH
PF2	315	0.03	**2.28**	27.76	0.39	17.73	0.59	2.7	7.31	37.49	1.989	0.04	11	7.78
PF3	244	0	1.97	22.78	0.39	8.09	0.17	1.85	8.19	30.19	1.217	0.03	3.6	7.56
PF4	225	0.05	1.35	10.76	0.39	5.14	0.1	1.53	2.7	33.25	1.122	0.03	5.8	7.45
PF5	433	0.06	**2.18**	15.17	0.45	20.81	0.52	4.08	29.18	43.67	2.998	0.06	6.1	7.31
PF6	577	0.12	1.83	20.8	0.5	31.37	1.06	9.3	35.02	58.38	13.76	**0.87**	41.2	8.41
PF8	233	0	1.50	17.06	0.38	4.77	0.35	1.37	7.85	34.41	1.212	0.01	1	7.27
PF9	291	0.02	**2.12**	28.01	0.4	14.85	0.48	2.37	10.68	33.8	1.337	0.01	5.7	7.43
PF10	369	**0.88**	1.22	20.8	0.43	26.61	0.76	2.55	18.86	2.15	2.156	0.04	11.8	7.44
PF12	265	0.04	**2.13**	27.5	0.39	14.96	0.48	2.43	10.82	30.11	1.428	0.03	7.2	7.41
PF13	332	0.10	1.43	23.86	0.4	10.56	0.26	3.16	8.31	43.08	3.589	0.09	16.3	7.89
PF14	459	0.23	1.55	17.02	0.39	15.87	0.54	2.74	19.52	51.54	3.801	0.03	16.7	7.3
PF15	369	0.19	1.42	6.71	0.36	5.23	0.71	3.89	11.23	36.23	1.416	0.04	8	7.55
PF22	399	0.30	**2.70**	14.78	0.43	33.08	1.15	2.55	15.53	38.11	2.903	0.04	11	7.38
PF23	492	**3.76**	1.65	15.63	0.5	52.31	1.54	7.04	18.07	34.48	9.176	**1.64**	42.3	7.51
PF25	374	0.07	1.65	22.48	0.41	11.35	0.46	3.69	9.7	45.56	1.17	0.04	0.4	7.58
PF26	522	0.40	1.23	18.42	0.53	10.64	1.28	2.35	27.66	47.39	1.855	0.06	9.1	7.42
DF7	1046	0	**2.92**	113.3	0.38	194.48	5.08	0.4	20.7	119.4	2.139	0.04	4.9	7.32
PS1	74,4	0.06	1.57	9.53	0.88	4.38	0.1	1.71	3.31	11,00	2.772	0.03	9.3	8.88
PS2	315	0.01	1.12	15.6	0.5	15.6	0.3	1.8	18.33	47.36	4.841	0.07	18.7	6.892
PS4	219	1.03	1.76	10.9	0.53	30.35	0.77	6.5	4.22	21.31	9.807	0.09	43.6	8.339
PS5	281	0.09	1.71	6.3	0.37	3.83	0.3	1.4	16.7	37.08	1.147	0.03	3.6	7.37

Table S3 Correlation matrix describing the relationship between *S. parasitica* load (response variable) and physico-chemical parameters of water (explanatory variables).

**Variables**	**pH**	**EC**	**NH4**	**NO3**	**SO4**	**F**	**Cl**	**Na**	**K**	**Mg**	**Ca**	**TOC**	**TP**	**COD**	***S. parasitica* load**
pH	**1**	-0.261	0.033	-0.025	-0.165	0.680	-0.113	-0.136	0.449	-0.214	-0.273	0.460	0.165	0.419	-0.202
EC	-0.261	**1**	0.111	0.445	0.791	-0.270	0.855	0.907	0.089	0.633	0.869	0.186	0.245	0.102	0.489
NH4	0.033	0.111	**1**	-0.134	-0.132	0.147	0.135	0.152	0.505	0.097	-0.182	0.475	0.831	0.632	-0.205
NO3	-0.025	0.445	-0.134	**1**	0.602	-0.205	0.598	0.557	-0.077	0.084	0.489	-0.050	-0.053	-0.126	0.350
SO4	-0.165	0.791	-0.132	0.602	**1**	-0.215	0.921	0.889	-0.278	0.167	0.804	-0.106	-0.072	-0.168	0.450
F	0.680	-0.270	0.147	-0.205	-0.215	**1**	-0.109	-0.119	0.150	-0.051	-0.302	0.296	0.155	0.281	-0.327
Cl	-0.113	0.855	0.135	0.598	0.921	-0.109	**1**	0.973	-0.076	0.269	0.782	0.127	0.156	0.088	0.442
Na	-0.136	0.907	0.152	0.557	0.889	-0.119	0.973	**1**	-0.055	0.353	0.800	0.109	0.172	0.073	0.477
K	0.449	0.089	0.505	-0.077	-0.278	0.150	-0.076	-0.055	**1**	0.366	-0.094	0.882	0.713	0.840	-0.262
Mg	-0.214	0.633	0.097	0.084	0.167	-0.051	0.269	0.353	0.366	**1**	0.424	0.394	0.345	0.246	0.016
Ca	-0.273	0.869	-0.182	0.489	0.804	-0.302	0.782	0.800	-0.094	0.424	**1**	0.063	0.043	-0.063	0.561
TOC	0.460	0.186	0.475	-0.050	-0.106	0.296	0.127	0.109	0.882	0.394	0.063	**1**	0.704	0.949	-0.267
TP	0.165	0.245	0.831	-0.053	-0.072	0.155	0.156	0.172	0.713	0.345	0.043	0.704	**1**	0.716	-0.228
COD	0.419	0.102	0.632	-0.126	-0.168	0.281	0.088	0.073	0.840	0.246	-0.063	0.949	0.716	**1**	-0.274
S. parasitica load	-0.202	0.489	-0.205	0.350	0.450	-0.347	0.442	0.477	-0.262	0.016	0.561	-0.267	-0.228	-0.274	**1**

**Color intensity of the correlation**	
	0 - 0.33 low positive correlation
	0.34 - 0.66 medium positive correlation
	0.67 - 1 strong positive correlation
	0 - (-0.33) low negative correlation
	-0.34 - (-0.66) medium negative correlation
	-0.67 - (-1) strong negative correlation

Table S4 Trout swab samples.

Code	Species	Animal status	Location	HTRS96 coordinate system		Sampling period	Copies per ul of original sample	DNA concentration (ng/uL)	*Saprolegnia parasitica* ITS copies/ng of total DNA
				x	y				
Z1	*Oncorhynchus mykiss* (Walbaum, 1792), rainbow trout	adult, healthy	Solin	499371	4821971	November 2018	3.9	0.34	11
Z2		adult, healthy					13.8	25	0.6
Z3		adult, healthy					3.4	12	0.3
Z4		adult, healthy					1.4	8.10	0.2
Z5		adult, healthy					4.2	6.20	0.7
Z6		adult, healthy					0.72	0.30	2
B4		adult, with injuries and/or skin lesions					1.38	0.74	2
J1		eggs, analysed directly from the farms					0.55	8.1	0.1
J2		eggs, analysed directly from the farms					0	0.89	0
J4		eggs, analysed directly from the farms					0	12	0
J5		eggs, analysed directly from the farms					0.26	0.53	0.5
J6		eggs, analysed directly from the farms					3.1	0.45	7
Z13		adult, healthy	Radovan	494270	5134418	January 2019	7.43	29	0.3
Z14		adult, healthy					0	11	0
Z15		adult, healthy					6.6	4.67	1
Z16		adult, healthy					1.1	70	0
Z17		adult, healthy					0.2	32	0
B10		adult, with injuries and/or skin lesions					0	2.28	0
B11		adult, with injuries and/or skin lesions					539688	4.57	118094
ZJ4		eggs, negative control in a laboratory infection trial					0	0.76	0
ZJ5		eggs, negative control in a laboratory infection trial					14.3	1.56	9
ZJ6		eggs, negative control in a laboratory infection trial					8.3	4.09	2
BJ5		eggs, infected with *Saprolegnia parasitica* in a laboratory infection trial					5216	0.42	12419
BJ6		eggs, infected with *Saprolegnia parasitica* in a laboratory infection trial					46640	2,57	18148

R2		adult, with injuries and/or skin lesions	Gračani	508709	5143992	December 2019	1359	3.03	449
R3		adult, with injuries and/or skin lesions					4977.5	71	70
R4		adult, with injuries and/or skin lesions					473	11	43
R5		adult, with injuries and/or skin lesions					151.8	5.2	29
R7		adult, with injuries and/or skin lesions					324.5	4.25	76
R8		adult, with injuries and/or skin lesions					161.7	3.85	42
R9		adult, with injuries and/or skin lesions					82.5	3.5	24
Z7	*Salmo trutta* (Linnaeus, 1758), brown trout	adult, healthy	Kostanjevac	420850	5064594	December 2018	11.18	34	0.3
Z8		adult, healthy					10.45	38	0.3
Z9		adult, healthy					1.7	5.8	0.3
Z10		adult, healthy					0	13	0
Z11		adult, healthy					1.4	23	0.1
Z12		adult, healthy					2	8	0.3
B6		adult, with injuries and/or skin lesions					24.2	0.26	93
B7		adult, with injuries and/or skin lesions					263.5	3.22	82
B8		adult, with injuries and/or skin lesions					0.37	1.44	0.3
BJ1		eggs, infected with *Saprolegnia parasitica* in a laboratory infection trial					371.2	2.94	126
BJ2		eggs, infected with *Saprolegnia parasitica* in a laboratory infection trial					76.5	0.35	219
BJ3		eggs, infected with *Saprolegnia parasitica* in a laboratory infection trial					35.8	0.57	63
ZJ1		eggs, negative control in a laboratory infection trial					0	7	0
ZJ2		eggs, negative control in a laboratory infection trial					0	1.06	0

Table S5 List of ITS sequences used to design *Saprolegnia parasitica*-specific primers.

Species	Acc. No.
Saprolegnia diclina	KF717745
Saprolegnia diclina	KF717795
Saprolegnia diclina	KF717797
Saprolegnia diclina	KF717816
Saprolegnia sp. (1)	KF717835
Saprolegnia sp. (1)	KF717836
Saprolegnia parasitica	KF717839
Saprolegnia parasitica	KF717842
Saprolegnia parasitica	KF717846
Saprolegnia parasitica	KF717854
Saprolegnia parasitica	KF717855
Saprolegnia parasitica	KF717860
Saprolegnia parasitica	KF717863
Saprolegnia parasitica	KF717864
Saprolegnia parasitica	KF717867
Saprolegnia parasitica	KF717869
Saprolegnia parasitica	KF717870
Saprolegnia parasitica	KF717872
Saprolegnia ferax	KF717883
Saprolegnia ferax	KF717958
Saprolegnia australis	KF717972
Saprolegnia australis	KF717974
Saprolegnia delica	KF718021
Saprolegnia delica	KF718022
Saprolegnia litoralis	KF718048
Saprolegnia sp. (2)	KF718049
Saprolegnia sp. (2)	KF718050
Saprolegnia sp. (2)	KF718055

Saprolegnia sp. (2)	KF718065
Saprolegnia sp. (2)	KF718071
Saprolegnia sp. (2)	KF718099
Saprolegnia sp. (3)	KF718122
Saprolegnia subterranea	KF718124
Saprolegnia torulosa	KF718125
Saprolegnia monilifera	KF718130
Saprolegnia monilifera	KF718131
Saprolegnia terrestris	KF718134
Saprolegnia terrestris	KF718135
Saprolegnia eccentrica	KF718140
Saprolegnia eccentrica	KF718141
Saprolegnia sp. (4)	KF718142
Saprolegnia furcata	KF718143
Saprolegnia sp. (5)	KF718144
Saprolegnia sp. (5)	KF718146
Saprolegnia sp. (5)	KF718152
Saprolegnia sp. (5)	KF718156
Saprolegnia sp. (6)	KF718174
Saprolegnia sp. (6)	KF718177
Saprolegnia asterophora	KF718178
Saprolegnia sp. (7)	KF718179
Saprolegnia megasperma	KF718186
Saprolegnia megasperma	KF718187
Saprolegnia turfosa	KF718190
Saprolegnia anisospora	KF718192
Leptolegnia sp.	KF718185
Protoachlya paradoxa	KF718202
Achlya caroliniana	KF718203
Aphanomyces astaci	KF718204
